# *SLC4A11* depletion impairs NRF2 mediated antioxidant signaling and increases reactive oxygen species in human corneal endothelial cells during oxidative stress

**DOI:** 10.1038/s41598-017-03654-4

**Published:** 2017-06-22

**Authors:** Sanjukta Guha, Sunita Chaurasia, Charanya Ramachandran, Sanhita Roy

**Affiliations:** 1 Prof. Brien Holden Eye Research Center, LVPEI, Hyderabad, India; 2 Tej Kohli Cornea Institute, LVPEI, Hyderabad, India; 3 Research Scholar, Manipal University, Manipal, India

## Abstract

Corneal endothelial dystrophy is a progressive disease with gradual loss of vision and characterized by degeneration and dysfunction of corneal endothelial cells. Mutations in *SLC4A11*, a Na^+^ dependent OH^−^ transporter, cause congenital hereditary endothelial dystrophy (CHED) and Fuchs’ endothelial corneal dystrophy (FECD), the two most common forms of endothelial degeneration. Along with genetic factors, oxidative stress plays a role in pathogenesis of several corneal diseases. In this study we looked into the role of *SLC4A11* in antioxidant stress response in human corneal endothelial cells (HCEnC). We found increased expression of *SLC4A11* in presence of oxidative stress. Depletion of *SLC4A11* using targeted siRNA, caused an increase in reactive oxygen species, cytochrome c, lowered mitochondrial membrane potential, and reduced cell viability during oxidative stress. Moreover, *SLC4A11* was found to be necessary for NRF2 mediated antioxidant gene expression in HCEnC. On the other hand, over expression of *SLC4A11* reduces reactive oxygen species levels and increases cell viability. Lastly, CHED tissue specimens show evidence of oxidative stress and reduced expression of NRF2. In conclusion, our data suggests a possible role of SLC4A11 in regulating oxidative stress, and might be responsible for both the etiology and treatment of corneal endothelial dystrophy.

## Introduction

Corneal endothelial dystrophy results in degeneration and dysfunction of endothelial cells of the cornea, thickening of the Descemet’s membrane (DM) and is characterized by loss in endothelial cell density^1^. CHED and FECD are two major forms of corneal endothelial dystrophies that lead to progressive opacity of the cornea and gradual vision loss and are associated with mutations in *SLC4A11* gene^2–4^. SLC4A11 is a 100 kDa transmembrane protein and although earlier thought to be a borate transporter^5^, recently has been shown to display Na^+^ coupled OH^−^ transport in bovine corneal endothelial cells^6, 7^. Lately, SLC4A11 has been identified as a NH_3_ or NH_3_:H+ co-transporter^8, 9^ and the cytoplasmic domain is absolutely essential for transport function of SLC4A11^10^. In addition to corneal endothelial dystrophy, mutations in *SLC4A11* also causes Harboyan syndrome^11, 12^, a form of progressive deafness. While mutations and loss of functional SLC4A11 are reported to be associated with degeneration and death of endothelial cells, the detailed physiological roles of SLC4A11 still remain unknown.

There is an increasing evidence to show that oxidative stress plays a significant role in the degeneration of the corneal endothelium and numerous other human diseases^13, 15^. The depletion of *SLC4A11* is seen to result in an increased apoptosis of human corneal endothelial cells^16^. Apoptosis has also been observed in corneal endothelial cells of Fuchs’ patients^17^. DNA damage in mitochondria and redox imbalance due to oxidative stress has also been reported in patients with FECD^18, 19^. In our earlier study, we have shown that cells expressing mutant SLC4A11 are more sensitive to oxidative stress mediated damages^20^. We therefore hypothesized that SLC4A11 may play a role in regulating oxidative stress.

Nuclear factor erythroid 2-related factor 2 (NRF2) plays an important role in regulating the redox potential and acts in defense mechanism against ROS. In response to oxidative stress, NRF2 provides cytoprotection to the cells and maintains redox homeostasis^21^. Under normal conditions it is held in the cytoplasm and tightly regulated by Keap1 that causes constant degradation of NRF2 by ubiquitination^22, 23^. On activation by oxidative stress and other external stimuli, it undergoes heterodimerization with small Maf proteins and translocates from the cytoplasm to the nucleus, where it binds to antioxidant responsive element^24^ and mediates transcription of its target genes which include various antioxidants and detoxification enzymes^21, 25, 26^. Some of the cytoprotective genes regulated by NRF2 are those of NAD(P)H-quinoneoxidoreductase 1 (NQO1), heme oxygenase 1 (HO-1) and glutathione reductase (GR)^27^.

In this study, we investigated the relationship between SLC4A11 and oxidative stress in both primary and immortalized HCEnC. Using siRNA to knockdown *SLC4A11* in HCEnC, we looked into the antioxidant signaling in response to oxidative stress in these cells. Our studies show that depletion of *SLC4A11* in corneal endothelial cells generates increased ROS, alters mitochondrial membrane potential and results in impaired NRF2 driven antioxidant signaling. Interestingly, CHED tissue specimens obtained from patients, also exhibit signs of oxidative stress and reduced NRF2 mediated antioxidant response. This study sheds light on physiological function of SLC4A11 during oxidative stress that can lead to the development of important noninvasive therapeutic interventions to prevent corneal endothelial degeneration.

## Results

### Oxidative stress up-regulates *SLC4A11* expression in HCEn and HEK 293 cells

**Figure 1:**
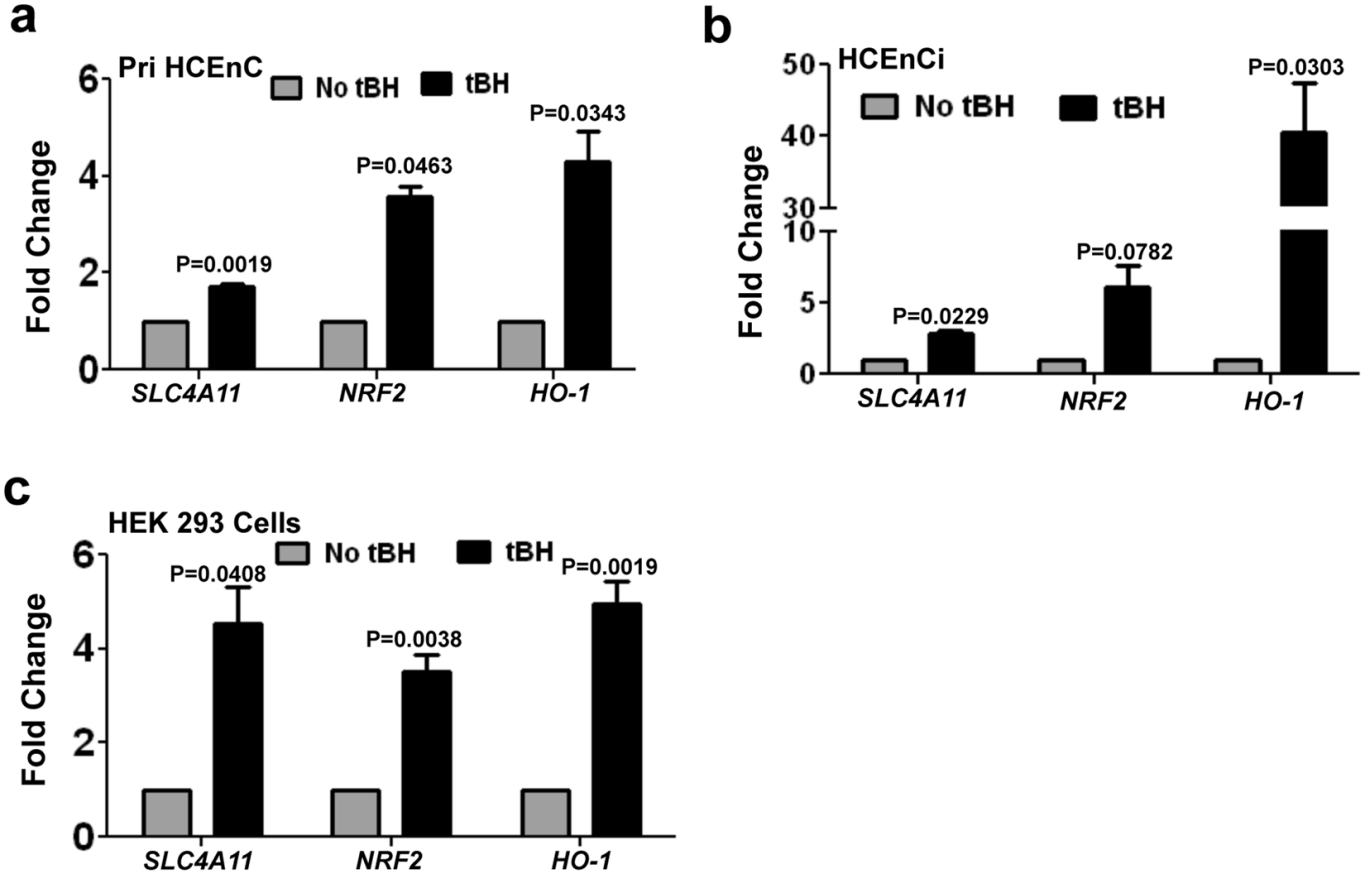
Up-regulation of *SLC4A11* in response to oxidative stress. Both primary (**a**), and immortalized (**b**) human corneal endothelial cells and HEK 293 (**c**) cells were exposed to 500 μM tBH for 4 h and fold changes of the expression of *SLC4A11*, *NRF2* and *HO-1* were determined by quantitative PCR. These experiments were performed three times.

Oxidative stress has been associated with pathogenesis of corneal endothelial dystrophy^28^ and other corneal diseases^29^. We have earlier reported that cells expressing mutant SLC4A11 are more prone to oxidative stress^20^ compared to cells expressing the wild-type protein. Thus we asked whether *SLC4A11* gene itself responds to oxidative stress. We exposed HCEnC, both primary and immortalized cells, and HEK 293 cells to 500 μM of tBH as exogenous source of oxidative stress over a period of 4 h. As shown in Fig. 1, there was a significant increase in the expression of *SLC4A11* and *NRF2* in both primary (A) and immortalized (B) human corneal endothelial cells. Oxidative stress also induced expression of *HO-1*, an antioxidant gene regulated by NRF2, by nearly 4 folds in primary cells (Fig. 1a) and about 40 folds in the immortalized cells (Fig. 1b). Transcripts of *SLC4A11*, *NRF2* and *HO-1* were also significantly induced in HEK 293 cells by tBH (Fig. 1c). To test that increase of *SLC4A11* expression is not tBH specific, we challenged HEK 293 cells with selenite (SN, 10 μM), as an alternative source of oxidative stress^30^. As seen in supplementary Figure S1, SN significantly increased the expression of *SLC4A11, NRF2 and HO-1*. Together these data indicate that *SLC4A11* is an oxidative stress response gene and might have a functional role in redox control.

### Depletion of *SLC4A11* causes increased ROS generation in HCEnC

**Figure 2:**
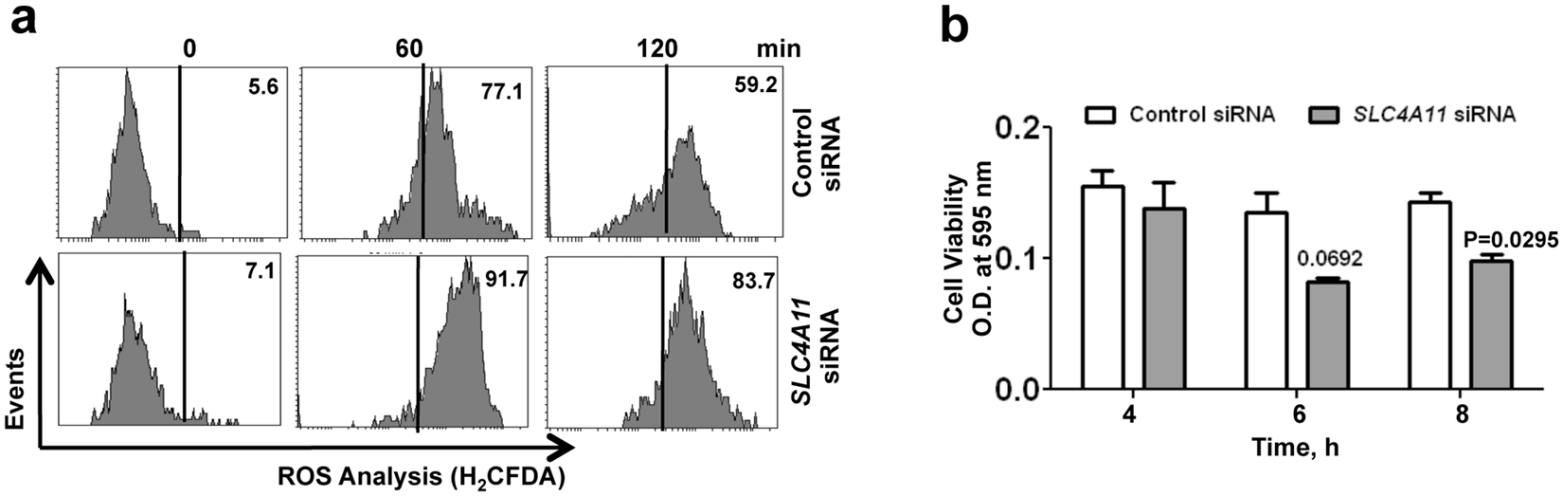
Determination of ROS and cell viability in *SLC4A11* depleted human corneal endothelial cells. ROS induced by oxidative stress was analyzed by flow cytometry using H_2_CFDA fluorescent probe. HCEnC were transfected with SLC4A11 targeted siRNA or control siRNA and exposed to 250 μM tBH, washed with 1X PBS, and ROS was determined (**a**). Cell viability was determined using MTT assay (**b**). Untreated cells were used as controls in each case. These experiments were performed twice.

Next we attempted to determine the link between SLC4A11 expression and ROS levels in corneal endothelial cells. In order to deplete the expression of *SLC4A11*, primary HCEnC were transfected using siRNA against *SLC4A11* and ROS was detected by flow cytometry using fluorescent ROS indicator H_2_CFDA. As shown in Fig. 2a, *SLC4A11* depletion causes increase in ROS positive cells by one hour (91.7%), compared to cells transfected with control-siRNA (77.1%). The difference became significant by 2 h where *SLC4A11*-siRNA transfected cells had higher ROS positive cells (83.7%) compared to that of control-siRNA transfected cells (59.2%). We further determined if *SLC4A11* depletion affects the viability of the cells in response to oxidative stress using MTT assays. Figure 2b shows that, cells depleted of *SLC4A11* had decreased viability at 4 and 6 h, which become significant by 8 h (p < 0.05). Together, these results demonstrate that depletion of *SLC4A11* expression induces intracellular ROS generation and decreases the viability of corneal endothelial cells in the presence of oxidative stress.

### Loss of *SLC4A11* alters mitochondrial membrane potential in HCEnC

**Figure 3:**
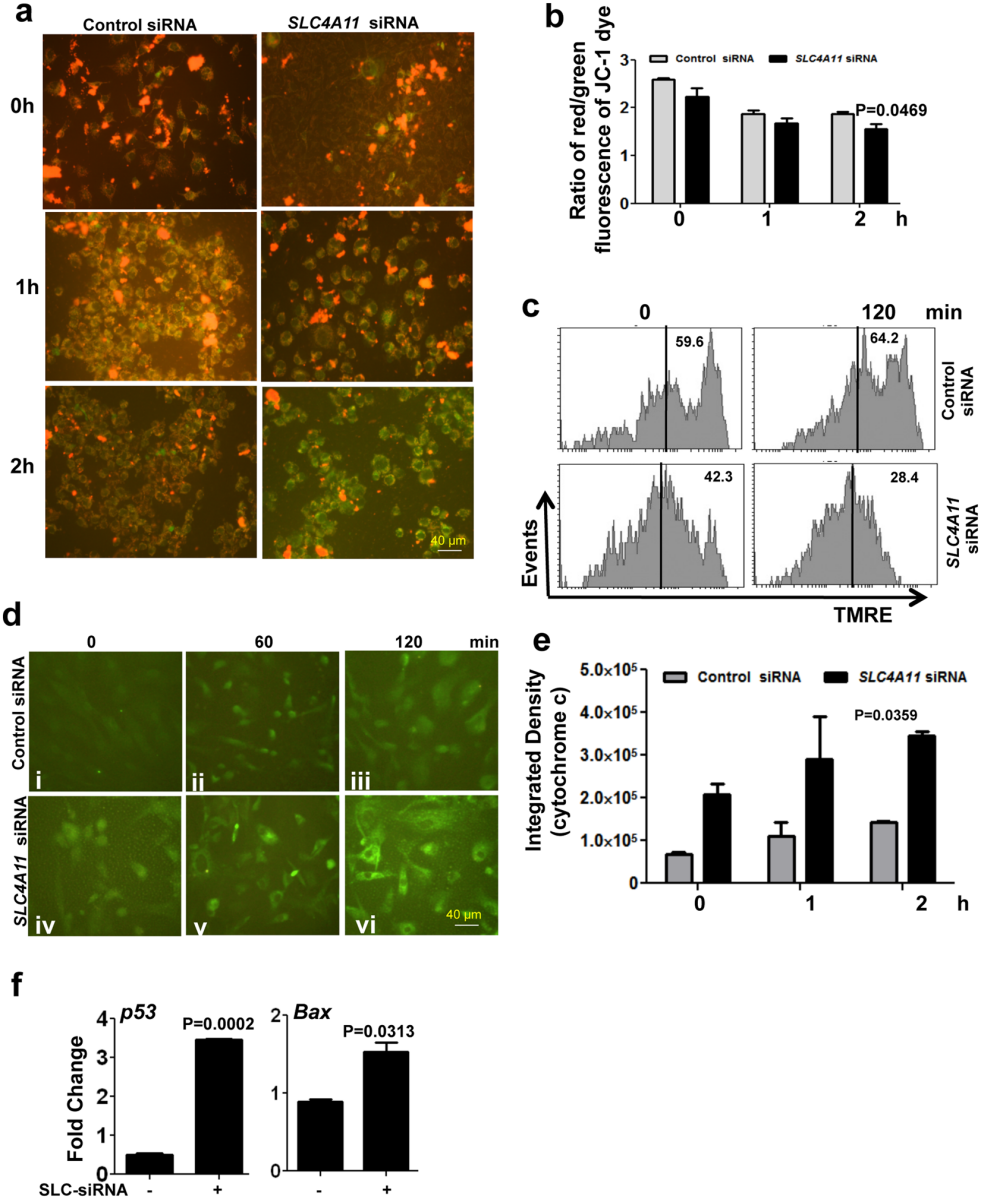
Mitochondrial activity in *SLC4A11* depleted human corneal endothelial cells exposed to oxidative stress. HCEnC were transfected with *SLC4A11* targeted siRNA or control siRNA and exposed to 250 μM tBH over 2 h, washed with 1X PBS, incubated with JC-1 and mitochondrial membrane potential was determined by microscopy (**a**), and ratio of red/green fluorescence intensities were quantitated (**b**). The mitochondrial membrane potential was also determined by flow cytometry after cells were incubated with TMRE following *SLC4A11* depletion and exposure to oxidative stress (**c**). The expressions of cytochrome c were imaged using fluorescence microscopy (**d**) and mean fluorescence intensities were quantitated (**e**). The expression of p53 and Bax genes was determined by quantitative PCR (**f**). Data are the representative of two different experiments, each performed with duplicates.

Oxidative stress is linked to mitochondrial dysfunction^31^, as mitochondria are both producers and targets of reactive oxygen species. To investigate whether depletion of *SLC4A11* affects the mitochondrial membrane potential, primary corneal endothelial cells transfected with control or *SLC4A11* siRNA were exposed to oxidative stress for 2 h. The cells were then incubated with the cationic dye JC-1, which localizes to normal mitochondrial membrane as red aggregates and turns into green monomer upon membrane depolarization. We found, using immunofluorescence, SLC4A11 depleted cells mostly green (depolarized) by 2 h compared to control-siRNA transfected cells in presence of oxidative stress **(**Fig. 3a **)**. The ratio of red/green intensity was determined quantitatively using ImageJ software^32^ and shows decreased ratio in *SLC4A11* depleted cells (Fig. 3b). We further checked the mitochondrial membrane potential of these cells by flow cytometry using TMRE, another cationic dye. TMRE is a mitochondria specific cell permeable dye that readily accumulates in active mitochondria and detects the mitochondrial membrane potential in live cells. Figure 3c shows that upon exposure to oxidative stress over 2 h, *SLC4A11* depleted cells have lower percentage of TMRE-positive cells (28.4%), suggesting a loss of mitochondrial membrane potential, in comparison to control cells (64.2%).

### Increased levels of cytochrome c and p53 in *SLC4A11* depleted HCEnC

When the trans-membrane potential of the mitochondria is affected, cytochrome c is released into the cytoplasm. The release of cytochrome c from the mitochondria initiates the apoptotic pathway in cells^33^. As there was a decrease in the mitochondrial trans-membrane potential in response to oxidative stress in *SLC4A11* depleted cells, we examined the level of cytochrome c in these cells on exposure to 250 µM tBH over 2 h. There was an increase in cytochrome c production by 1 h in cells depleted of *SLC4A11* (Fig. 3d v), in comparison to control cells (Fig. 3d ii), this became more significant by 2 h in *SLC4A11* depleted cells (Fig. 3d vi). The fluorescence intensity was quantitated using ImageJ software^32^ and the graph shows the significant reduction in the fluorescence between *SLC4A11* depleted and control cells (Fig. 3e). p53 and Bax gene expression was also determined in presence of oxidative stress, and Fig. 3f shows significant increase in p53 (4 fold) and Bax (1.5 fold) expression in *SLC4A11* depleted cells compared to control siRNA transfected cells.

### *SLC4A11* is required for NRF2 mediated antioxidant response in HCEnC

**Figure 4:**
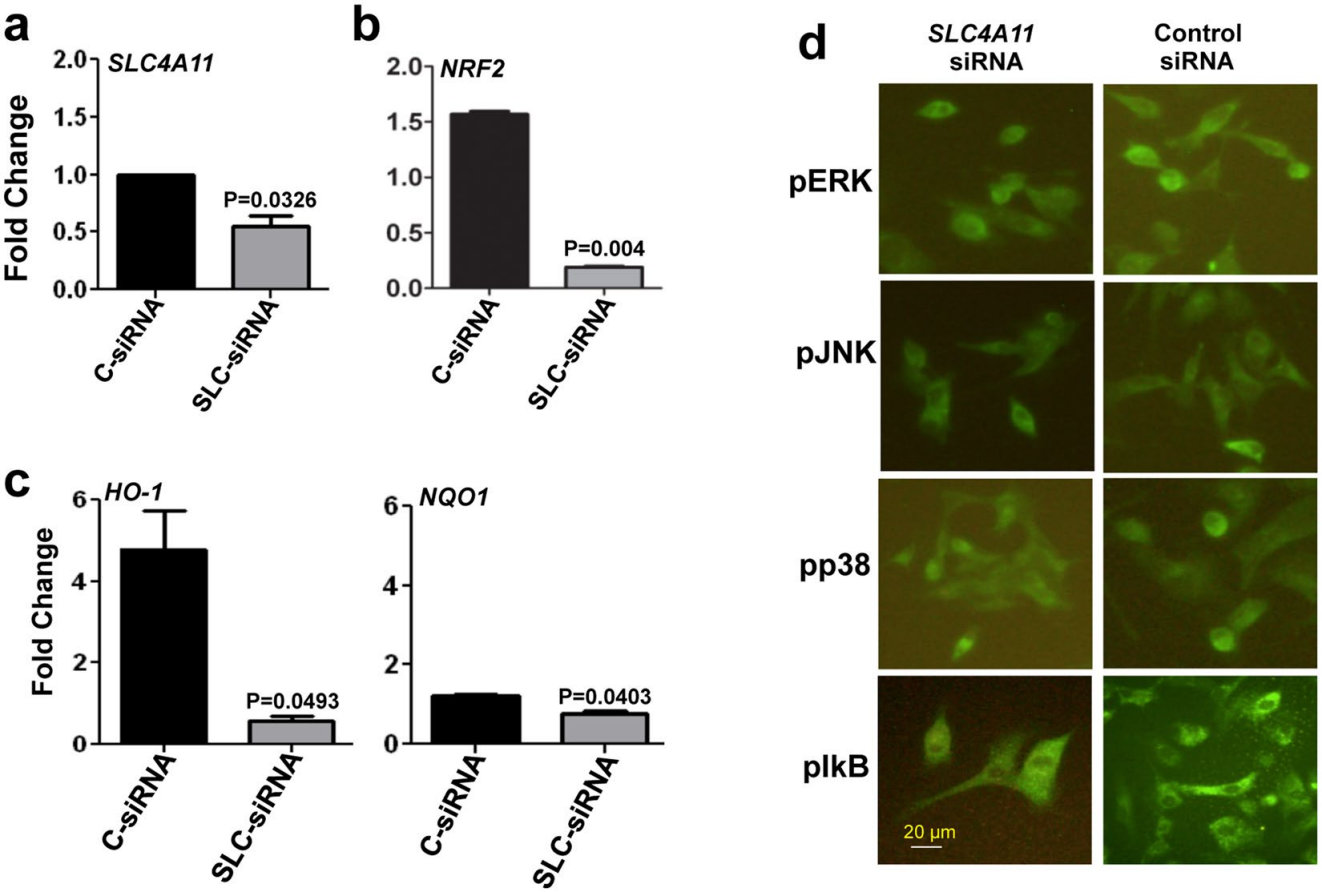
Depletion of *SLC4A11* impairs NRF2 mediated antioxidant responses in immortalized human corneal endothelial cells. HCENCi were transfected with *SLC4A11* siRNA (SLC-siRNA) or nonspecific control siRNA (C-siRNA) and exposed to 500 μM tBH for 4 h, and fold changes in gene expressions were determined by quantitative PCR. The depletion of *SLC4A11* (**a**), and changes in expression of *NRF2* (**b**), and antioxidant genes *HO-1*, and NQO1 (**c**) were examined. The activation of pIkB, pp38, pJNK and pERK was analyzed by immunocytochemistry (**d**). The experiments a, b, c was done three times in duplicates and experiment d was performed two times.

Oxidative stress is regulated by antioxidant signaling, and mediated by various genes whose expression depends on the transcription factor NRF2. Since expression of both *SLC4A11* and *NRF2* were up-regulated by oxidative stress (Fig. 1), we evaluated the NRF2 expression in *SLC4A11*-siRNA transfected cells exposed to oxidative stress. Immortalized human corneal endothelial cells were transfected with *SLC4A11*-siRNA and the level of *SLC4A11* was checked. A significant decrease in *SLC4A11* expression was found compared to cells transfected with control-siRNA (Fig. 4a). These cells were then exposed to 500 μM tBH for 4 h, and the gene expressions monitored. NRF2 expression was significantly lower in *SLC4A11* depleted cells, compared to control cells under oxidative stress conditions (Fig. 4b). Consistent with low NRF2 expression, *SLC4A11* depleted cells showed significantly reduced expression of NRF2 transcriptional target genes *HO-1*, and *NQO1*, compared with control cells under oxidative stress (Fig. 4c). The depletion of *SLC4A11*, however, did not affect the phosphorylation of the IkB and MAPK pathway proteins like ERK, JNK and p38 (Fig. 4d) in immortalized human corneal endothelial cells.

**Figure 5:**
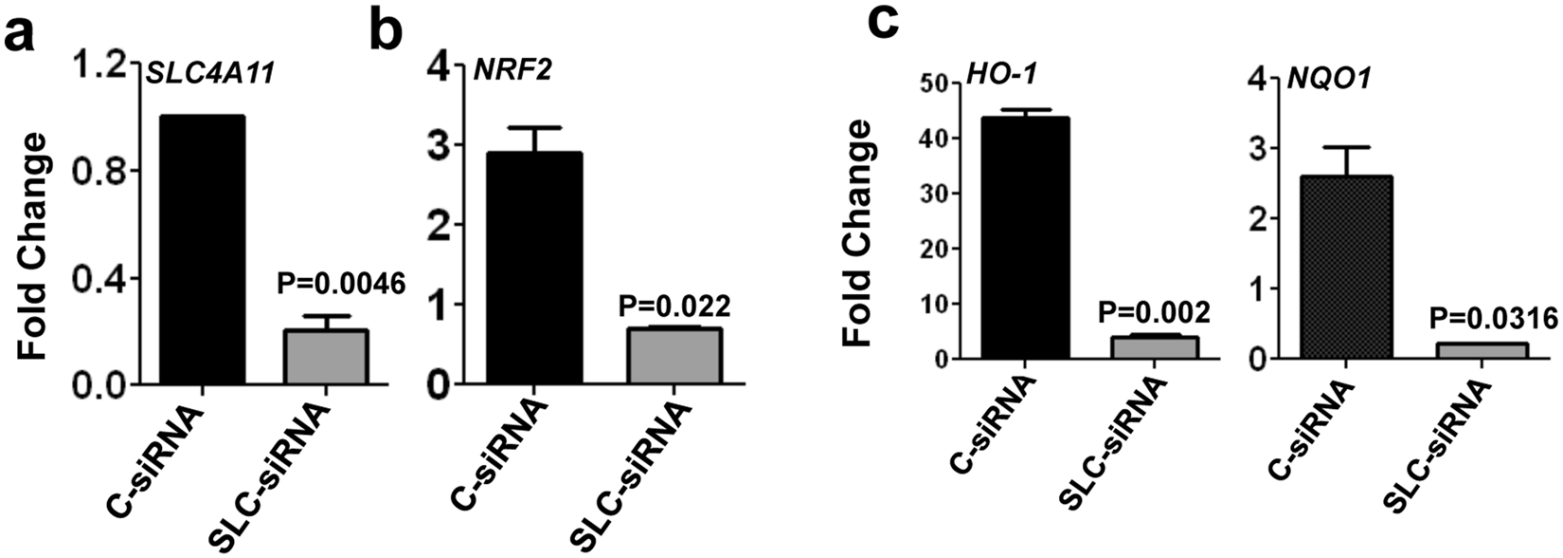
Depletion of *SLC4A11* impairs NRF2 mediated antioxidant responses in primary human corneal endothelial cells. Primary corneal endothelial cells were transfected with either *SLC4A11* siRNA (SLC-siRNA) or nonspecific control siRNA (C-siRNA) and exposed to 500 μM tBH for 4 h, and fold changes in gene expressions of *SLC4A11* (**a**), *NRF2* (**b**), and antioxidant genes *HO-1*, *NQO1* (**c**) were determined by quantitative PCR. These experiments were done three times in duplicates.

In order to ensure if primary corneal endothelial cells behave in a similar way, *SLC4A11* was depleted from primary cells using siRNA. The depletion of *SLC4A11* is confirmed as shown in Fig. 5a. Similar to immortalized cells, we found a significant decrease in expression of NRF2 in these *SLC4A11* depleted cells (Fig. 5b) The expressions of antioxidant genes were also checked and as shown in Fig. 5c, there were a significant decrease in expression of *HO-1*, and *NQO1*, in *SLC4A11*-siRNA transfected cells compared to control-siRNA transfected cells.

### *SLC4A11* overexpression increases cell viability, reduces ROS generation and protein nitration in cells in response to oxidative stress

**Figure 6:**
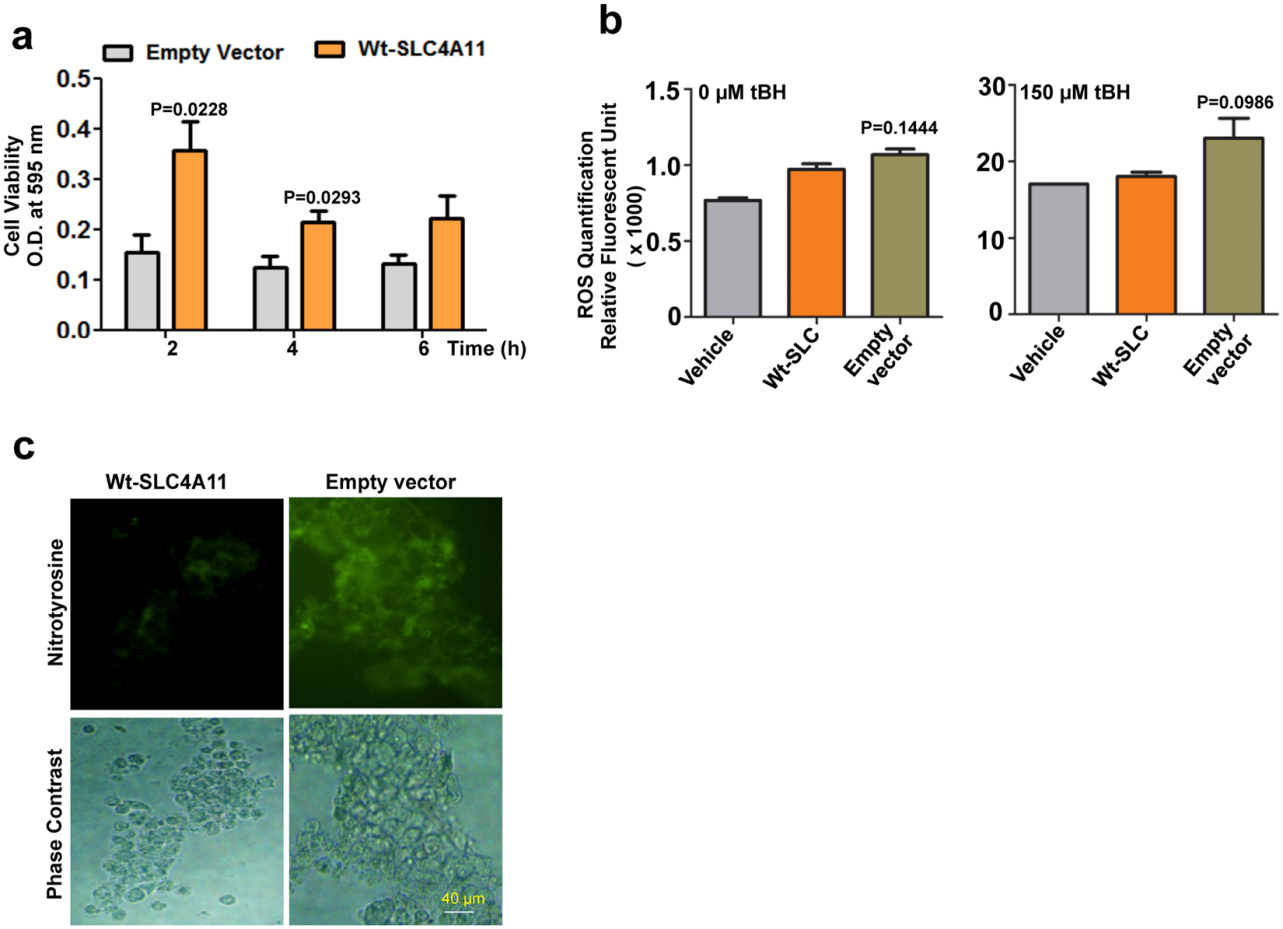
Overexpression of SLC4A11 in HEK 293 cells. Cells were transfected with wild-type. *SLC4A11* plasmid or empty vectors and exposed to 250 µM of tBH. Cell viability (**a**), ROS generation (**b**) and Nitrotyrosine staining (**c**) were determined. For nitrotyrosine staining, cells were stained using anti-nitrotyrosine antibody, followed by Alexafluor 488 secondary antibody. Cells were imaged both under bright field and fluorescence and images were captured by Olympus IX73 using 20X objective. The experiments a, and b, were done three times in duplicates and experiment c was performed two times.

As depletion of SLC4A11 causes a reduced expression of antioxidant genes, we wanted to determine the effects of SLC4A11 on the sensitivity of cells to oxidizing agents. In order to do so, HEK 293 cells were transfected with wt-SLC4A11 plasmids (wt-SLC) or empty vectors. For the overexpression assays, HEK 293 cells were used to minimize the effect of endogenous protein expression. The cells were exposed to 250 μM tBH or 10 μM SN for defined time periods, after which, the cell viability was determined using MTT assay. Cells transfected with wt-SLC were significantly more viable and resistant to tBH (Fig. 6a) or SN (Supplementary Fig. S2) mediated cell death while cells expressing empty vectors were not so. Next, we determined the effect of SLC4A11 overexpression on the ROS level by transfecting cells with wt-SLC or empty vectors and exposing them to 250 μM tBH for 2 h. The ROS level was detected by a quantitative assay of carboxy-DCF fluorescence. Non-transfected cells, treated in similar way, were used as the negative control. As shown in Fig. 6b, cells transfected with wt-SLC caused reduced levels of ROS, although not significant, compared to cells transfected with empty vectors in presence of oxidative stress. We then wanted to check if SLC4A11 overexpression could inhibit protein nitration in these cells. Cells transfected with wt-SLC or empty vectors were exposed to tBH for 2 h, washed and stained with anti-3-nitrotyrosine (3-NT) antibody and imaged under a fluorescent microscope. Cells transfected with the empty vector has increased expression of 3-NT as evident from Fig. 6c. Taken together, these data imply that overexpression of SLC4A11 protects cells against oxidative stress.

### Oxidative stress in CHED patients

**Figure 7:**
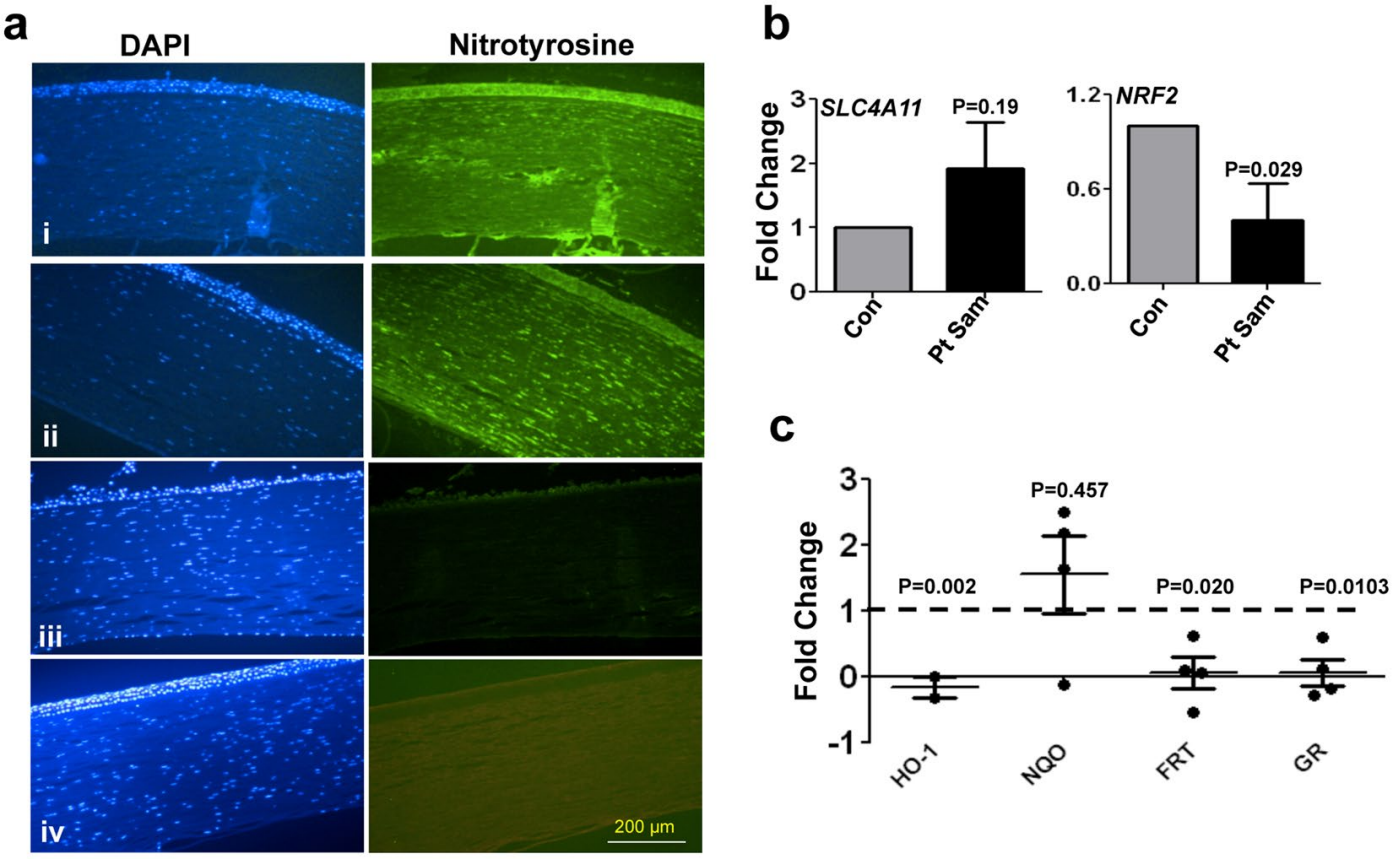
Oxidative stress in CHED tissue specimens. Tissue sections of CHED corneas (i, ii) and cadaveric corneas (iii, iv) were stained with anti-nitrotyrosine antibody, followed by Alexafluor 488 secondary antibody and were counterstained using DAPI. The sections were imaged under fluorescence microscope using 20X objective (**a**) (n = 5). The fold changes in the gene expression of *SLC4A11*, *NRF2* (**b**) and antioxidant genes *HO-1, NQO1, FRT* and *GR* (**c**) were determined from the DM-HCEnC complex obtained from CHED patients during DSEK procedures by quantitative PCR (n = 5). Data points represent individual patients.

Although presence of oxidative stress has been manifested in several corneal disorders including FECD and keratoconus^28, 34, 35^, there are no reports of the same for CHED. Our next experiments were on tissue specimens that were obtained from patients with CHED (n = 5), who underwent penetrating keratoplasty (PK) at our Institute. As shown in Fig. 7a, specific staining for 3-NT was observed in the corneal tissue specimens of the CHED patients (i, ii), while there was minimal or no staining for 3-NT in control cadaveric corneas (iii, iv). We next measured expression of oxidative stress markers in HCEnC-DM complex isolated from 5 CHED patients who underwent Descemet’s stripping endothelial keratoplasty (DSEK). While the expression of *SLC4A11* (mutated in these tissues) did not show a major change, there was significant reduction in expression of *NRF2* (Fig. 7b **)** accompanied by reduced expression of *HO-1*, *ferritin (FRT)*, *GR*, and to some extent of *NQO1* in CHED patients (Fig. 7c). Together, these data provide strong evidence for oxidative stress and reduced antioxidant genes level in CHED patients.

## Discussion

Oxidative stress has been increasingly reported to be involved in the pathogenesis for several ocular diseases^36^ including corneal endothelial dystrophies^13, 28^. There are earlier reports regarding oxidative stress induced degeneration of cells in corneal endothelial dystrophies. Although the incidence of CHED is low in western countries, in India it is accounted for 21 percent for all pediatric keratoplasties^37^. On the other hand, FECD is more common in North America and account for 10% of all corneal transplants^38^. The mutations in *SLC4A11* gene have been found associated for both FECD and CHED^2–4^. *SLC4A11* mutations cause dysfunction of endothelial cells leading to thickening of the DM and corneal edema. Recently, we found that cells expressing mutant SLC4A11 are more prone to oxidative stress^20^. *SLC4A11* null mice also exhibit signs of CHED such as corneal edema, swelling of endothelial cells and thickening of DM^39^.

In this study we report *SLC4A11* as an oxidative stress response gene required for proper NRF2 activation under oxidative stress conditions in human corneal endothelial cells. We found increased ROS level and decreased cell viability in SLC4A11 deficient cells in presence of oxidative stress. We have earlier reported increased accumulation of ROS in cells expressing mutant SLC4A11^20^. Higher levels of H_2_O_2_ were earlier detected in FECD corneal endothelial cells^40^. Liu *et al*. have previously reported decrease in cell viability in *SLC4A11*-shRNA transfected human corneal endothelial cells 7 to 10 days post transfection compared to control cells^16^. Although ROS is generated in response to various stimuli, increased levels of ROS can cause damage to DNA, lipids or proteins and is also an important contributor for mitochondrial dysfunction. In accordance to our earlier report of lowered mitochondrial membrane potential in cells expressing mutant *SLC4A11*, we found altered mitochondrial membrane potential in *SLC4A11* depleted cells as evident from green monomers formed from incubation with JC-1 dye. Altered mitochondrial structure and decreased activity were previously reported in corneal fibroblasts obtained from patients with type 2 granular corneal dystrophy^41^. High occurrence of mitochondrial DNA lesion was also found in the DM of FECD patients compared to the controls that indicate incompetent mechanism of removal of damaged mitochondrial DNA in FECD^18^. Increased cytochrome c, caspase activation and apoptotic cell death, are associated with loss of mitochondrial membrane potential. In our study, we detected increased cytochrome c expression along with increase in the expression of p53 and Bax, a pro apoptotic gene, in cells depleted of *SLC4A11*. This is in agreement with previous studies that showed increased level of p53^40^ and Bax^16^ in FECD and *SLC4A11* depleted corneal endothelial cells respectively. Increased caspase-3 activation in cells expressing mutant SLC4A11 has been reported earlier^20^ along with caspase 3, 7, and 9 in *SLC4A11* depleted human corneal endothelial cells^16^. p53 has been reported to play a role in antioxidant signaling by suppression of NRF2 activation and antioxidant gene expression^42^. Since NRF2 is the master regulator for antioxidant signaling^43^, we checked the expression of NRF2 and antioxidant genes in *SLC4A11* depleted cells. We saw significant reduction in the expression of NRF2 and NRF2 mediated antioxidant genes in cells depleted of *SLC4A11*. Bitar *et al*. have reported reduced NRF2 translocation and degradation of DJ-1, a NRF2 stabilizer, in FECD corneal endothelial cells^19^. We also observed decreased NRF2 and antioxidant gene expression in CHED tissue specimens which is similar to what has been observed for FECD^28^. Taken together, our results indicate a generalized down-regulation of the antioxidant genes in cells with depleted *SLC4A11* expression. This is important and gives an indication that inducing the expression of antioxidant genes might help in overcoming the oxidative stress in corneal endothelial cells. Very recently, there is a promising report where glafenine has been able to correct the trafficking defect of SLC411 mutants and increased its functionality^44^. *SLC4A11* was initially reported as a borate transporter and as borate has been shown to have mitogenic effect mediated by MAPK^5^, we checked if *SLC4A11* depletion has any effect on MAPK and NfkB pathways. Our results show that there was no difference in the activation of NF-κB and MAPK proteins in cells depleted of *SLC4A11* compared to control cells. There are earlier reports stating that over expression of *SLC4A11*, then known as NaBC1, in HeLa cells helps in cell proliferation in presence of borate^5^. We also over expressed SLC4A11 in HEK 293 cells and looked into the response of these cells towards oxidative stress and found that there was significant increase in cell viability and reduced ROS in these cells compared to control cells. SLC4A11 overexpression also reduced the levels of protein nitration in HEK 293 cells. The mechanism by which SLC4A11 regulates antioxidant signaling is in scope for future study and remains to be determined. We hypothesize that a positive regulatory feed-back loop might be present between SLC4A11 and NRF2 that helps to mobilize cell’s antioxidant machinery. SLC4A11 might regulate optimum activation of NRF2 in corneal endothelial cells by direct interaction or by promoting protein stabilization.

Increased oxidative stress is a prominent feature in corneal endothelial dystrophies, particularly for FECD. However, there is no direct evidence for the presence of oxidative stress in CHED which results from mutations of *SLC4A11*. We show here that increased oxidative stress is present in the corneal sections of CHED patients. Nitrotyrosine staining has been observed earlier in FECD and keratoconus corneas, but was absent in bullous keratopathy, although it has clinically similar signs as FECD^29^. To our knowledge, this makes our study to be the first to report the presence of oxidative stress in CHED. We also observed decreased expression of NRF2 and NRF2 target genes in CHED patients. Since *SLC4A11* is induced by oxidative stress, reduced NRF2 expression may explain presence of oxidative stress in CHED patients due to diminished or total loss of SLC4A11 functions owing to mutations of *SLC4A11*. Although there is significant reduction of a few antioxidant genes in CHED patients, we found that is not the case for NQO1 which suggest that there might be some redundant mechanism of NQO1 regulation involving other transcription factors^45^. Increased oxidative DNA damage, decreased expression of NRF2 and peroxiredoxins has also been reported in FECD tissues^46^. Although mutations in *SLC4A11* are associated with both CHED and FECD, one is autosomal recessive^2^ while the other is autosomal dominant disease^3^. Moreover, apart from *SLC4A11*, several other genes like *ZEB1* ^47^, *COL8A2* ^48^, *TCF4* ^49^, *LOXHD1* ^50^ have also found to be associated with FECD. Thus, defect in antioxidant signaling due to *SLC4A11* mutation might affect in a diverse pattern, which can also be the reason for delayed manifestation of the disease in FECD. It will be highly interesting to find out the extent of oxidative stress and difference in the expression of antioxidant gene between these two forms of corneal endothelial dystrophy.

Taken together, our study suggests a novel role of SLC4A11 in oxidative stress response in HCEnC. These findings can be made therapeutically beneficial to prevent or at least delay degeneration of corneal endothelium by enhancing antioxidant defenses and protecting the cells from reactive oxygen species. Currently, surgical interventions in form of DSEK or PK are the only modes of treatment for corneal endothelial dystrophy. In this context, our findings that deregulated NRF2 dependent antioxidant signaling due to *SLC4A11* depletion may provide new possibilities for noninvasive therapeutic interventions.

## Materials and Methods

### Tissue procurement

Tissue procurement for research purposes was approved by the Institutional Review Board of L.V. Prasad Eye Institute, Hyderabad, India and the research followed the tenets of the Declaration of Helsinki. The tissue specimens were collected after obtaining informed consent from the patients.

### Culture of primary human corneal endothelial cells

Primary corneal endothelial cells were cultured from human donor corneas sourced from the Ramayamma International Eye Bank (LV Prasad Eye Institute, Hyderabad, TS). Briefly, the DM was carefully peeled, chopped into smaller pieces and subjected to collagenase (1 mg/ml, Sigma, St. Louis, MO) digestion for 2 h at 37 °C. The cells were collected by centrifugation following trypsin-EDTA (0.25%) treatment for 5 min. The cells were cultured on FNC (Athena, Baltimore, MD) pre-coated plastic dishes in medium containing Opti-MEM, 8% FBS, 0.08% chondroitin sulphate, 200 mg/L calcium chloride, 5 ng/ml hEGF and 1X antibiotic/antimycotic. Cells were cultured until they reached confluence and were split using trypsin-EDTA^51^. Passage 1–3 cells were used for further experiments.

### Immortalized human corneal endothelial cells

The characterized corneal endothelial cell line (HCEnCi) was a kind gift from Dr. Ula Jurkunas (Schepens Eye Research Institute, USA)^52^. The cells were cultured in FNC coated plastic dishes in medium similar to the primary cells but with the addition of bovine pituitary extract (100 μg/ml, Lonza, Walkersville, MD) at 37 °C with 5% CO_2_. Once confluent, the cells were sub-cultured using 0.25% trypsin-EDTA.

### Oxidative Stress

The oxidative stress was initiated by exposing the human corneal endothelial cells to different concentrations of tert-butyl hydroperoxide (tBH; Sigma-Aldrich, St. Louis, MO) or sodium selenite (SN; Sigma-Aldrich, St. Louis, MO) diluted in serum free cell culture media for different time points as mentioned in the text. Controls were incubated with media alone.

### RNA Isolation and Quantitative PCR

Quantitative real time PCR (QPCR) was done to evaluate mRNA expression of *SLC4A11*, *NRF2*, and several other genes in human corneal endothelial cells. Total RNA was isolated using TRIzol (Invitrogen, Carlsbad, CA) and was reverse transcribed using cDNA synthesis kit according to the manufacturer’s details (Eurogentec, Belgium). QPCR was performed on ABI 7900HT (Applied Biosystems, Foster City, CA) using the Sybr green PCR master mix (Thermo Fisher, Waltham, MA). Relative quantities of mRNA expression of different genes were normalized using the 2^−ΔΔCt^ method using *18 s rRNA* as the housekeeping gene. The sequences of the primers used are in Table 1.

**Table 1:** List of primers.

Gene	Sequence (5′ → 3′)	
SLC4A11	FWD:ATGTCGCAGAATGGATACTTCG	REV:AAAACGGATACTCTCGCCAG
NRF2	FWD:CGGTATGCAACAGGACATTG	REV:ACTGGTTGGGGTCTTCTGTG
FR	FWD:CCCCCATTTGTGTGACTTCAT	REV:GCCCGAGGCTTAGCTTTCATT
HO-1	FWD:AAGACTGCGTTCCTGCTCAAC	REV:AAAGCCCTACAGCAACTGTCG
NQO1	FWD:GAAGCCGCAGACCTTGTGAT	REV:CTGCCTTCTTACTCCGGAAGG
GR1	FWD:CACGAGTGATCCCAAGCCC	REV:CAATGTAACCTGCACCAACAATG
18 S RNA	FWD:GGAGTATGGTTGCAAAGCTGA	REV:ATCTGTCAATCCTGTCCGTGT
P53	FWD:AACGGTACTCCGCCACC	REV:CGTGTCACCGTCGTGGA
Bax	FWD:CCCGAGAGGTCTTTTTCCGAG	REV:CCAGCCCATGATGGTTCTGAT

### RNA Interference Knockdown of SLC4A11

siRNA was used against human *SLC4A11* for RNA interference in human corneal endothelial cells. Non-targeted scrambled siRNA was used as control. All siRNAs were reconstituted to a final concentration of 50 μM according to the manufacturer’s protocol (Santa Cruz, Dallas, TX). Cells were grown overnight, washed in 1X PBS and transfected with 60 nM of siRNA using Lipofectamine 3000 TM transfection agent (Invitrogen, Carlsbad, CA), according to manufacturer’s protocol.

### Transfection of HEK 293 cells

HEK 293 cells were grown in DMEM media (Invitrogen, Carlsbad, CA) supplemented with 5% heat-inactivated fetal bovine serum and 0.5% penicillin streptomycin at 37 °C. Cells were transiently transfected with *SLC4A11* plasmid or empty vectors using lipofectamine 3000 as descried earlier^20^. The wild-type *SLC4A11* plasmid was a kind gift from Dr. J.A. Bonanno, Indiana University, USA^6^.

### Determination of reactive oxygen species by flow cytometry

For intracellular ROS determination in HCEnC, transfected cells exposed to tBH or media for defined time periods were washed in 1X PBS and incubated with 10 μM dicholorodihydrofluoresceindiacetate (H_2_CFDA; Invitrogen) for 20 min in dark at 37 °C. Cells were further washed in 1X PBS, and immediately analyzed by flow cytometry using BD Facsaria II (BD Biosciences, San Jose, CA).

### Immunocytochemistry analysis

For immunocytochemistry analysis, 1 × 10^4^ human corneal endothelial cells were seeded on coverslips and transfected with control or *SLC4A11* siRNA as described above. The cells were then exposed to oxidative stress, washed in PBS and fixed in 4% paraformaldehyde for 15 min. For immunofluorescence analysis of pIkB, MAPK, Nitrotyrosine, or cytochrome c, staining was done as described^53^. Cells were stained with rabbit anti-pERK, anti-pJNK, anti-pp38 or anti-pIkB antibodies (1:100, Cell Signaling Technology, Beverly, MA), mouse anti-nitrotyrosine (1:100, Novus Biologicals, Littleton, CO), or mouse anti-cytochrome c antibody (1:100, BioLegend, SanDiego, CA) for 45 min followed by Alexafluor 488 secondary antibody (1:500, Molecular probes, Eugene, OR) for 30 min. Images were captured on a fluorescent microscope (Olympus IX73) using a 20X objective. The fluorescence intensities were measured by ImageJ software.

### Determination of mitochondrial membrane potential

Primary human corneal endothelial cells were cultured overnight on coverslips, transfected with control or *SLC4A11* siRNA and exposed to 250 μM of tBH for defined time periods. The cells were then washed with 1X PBS and incubated with cationic dye JC-1 (final concentration 2 μM; Santa Cruz, Dallas, TX) for 20 min at 37 °C. The cells were washed with 1X PBS and coverslips were inverted onto slides and live cells were imaged to detect JC-1 staining under fluorescent microscope (Olympus IX73) using 20X objective. All images were acquired at equal exposure time to compare the fluorescence. The red and green fluorescence intensities of the images were measured by ImageJ using ROI Manager tool. To analyze the mitochondrial membrane potential by flow cytometry, cells after being exposed to oxidative stress were incubated with tetramethylrhodamine ethyl ester (TMRE; final concentration 200 nM; Santa Cruz, Dallas, TX) for 20 min at 37 °C and fluorescence was determined by flow cytometry using BD Facsaria II (BD Biosciences, San Jose, CA).

### Cell viability Assay

Cell viability was determined quantitatively using a 3-[4,5-Dimethylthiazole-2yl]-2,5-diphenyltetrazolium bromide (MTT) (Invitrogen) cleavage assay. In brief, 1 × 10^4^ cells were plated in 96 well plates and transfected with *SLC4A11-*siRNA, control siRNA or *SLC4A11* plasmid, empty vectors. Cells were exposed to tBH or SN as mentioned above. After washing with 1x PBS, cells were incubated with 5 mg MTT per ml of culture media for 2 h at 37 °C. The supernatant was discarded and stop solution (DMSO) was added to each well to dissolve the formazone crystals. Absorbance was measured at 595 nm in SpectraMax M3 Reader (Molecular Device).

### Nitrotyrosine Staining of Tissues

Archived formalin fixed, paraffin embedded corneas with confirmed clinical diagnosis of CHED (n = 5) were obtained along with three normal cadaveric corneas unsuitable for transplantation. 5 μm sections were mounted on slides and were deparafinized, hydrated and permeabilized, then rinsed with 1X PBS. The sections were incubated with mouse anti-3 nitrotyrosine antibody (1:50, Novus Biologicals, Littleton, CO), for 1 h, washed and reincubated with Alexafluor 488 secondary antibody (1:250, Molecular probes, Eugene, OR) for 1 h and further washed. The sections were counterstained with DAPI (Abcam, Cambridge) and observed under fluorescent microscope (Olympus IX73) using 20X objective and imaged using Olympus DP71 camera.

### Statistics

Statistical analysis was performed using either an unpaired t test or one way Anova (Prism, GraphPad Software). *p* values less than 0.05 was considered significant.

## Electronic supplementary material

Supplementary Info
