# Supplementary material for: SLC4A11 depletion impairs NRF2 mediated antioxidant signaling and increases reactive oxygen species in human corneal endothelial cells during oxidative stress
Source: Sci Rep. 2017 Jun 22;7:4074. doi: 10.1038/s41598-017-03654-4 (PMC5481427; doi:10.1038/s41598-017-03654-4)
Supplement: Supplementary file 1 — Supplementary Info [file 41598_2017_3654_MOESM1_ESM.pdf]

***SLC4A11* depletion impairs NRF2 mediated antioxidant signaling and increases reactive oxygen species in human corneal endothelial cells during oxidative stress**

Sanjukta Guha[1,2], Sunita Chaurasia[2], Charanya Ramachandran[1,2], Sanhita Roy[1,2]*

[1] Prof. Brien Holden Eye Research Center, Hyderabad, India

[2] Tej Kohli Cornea Institute, Hyderabad, India

Supplementary Information

Supplementary Figure 1

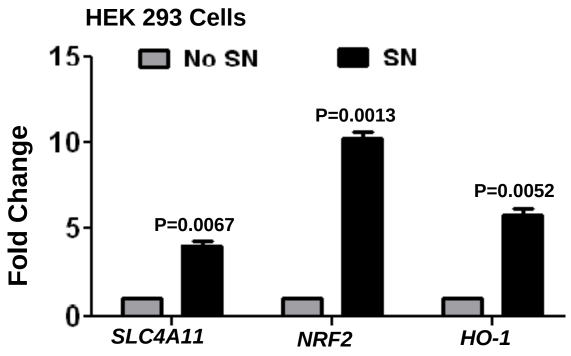

Supplementary Fig. 1: Selenite induces expression of *SLC4A11* and antioxidant genes. HEK 293 cells were exposed to 10 μM selenite for 4 h and fold changes of the expression of *SLC4A11, NRF2* and *HO-1* were determined by quantitative PCR.

Supplementary Figure 2

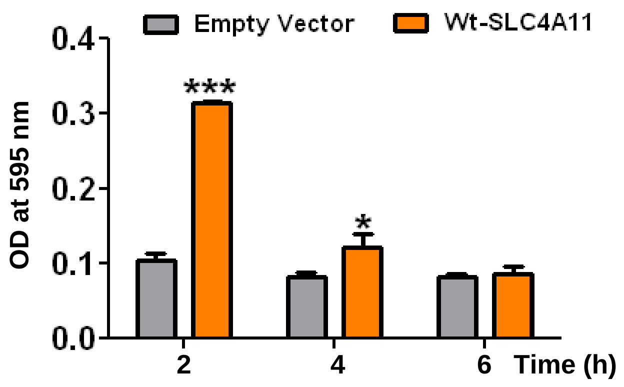

Supplementary Fig. 2: Overexpression of SLC4A11 increases cell viability against oxidative stress. Cells were transfected with wild-type *SLC4A11* plasmid or empty vectors and exposed to 10 μM of SN for 2, 4 and 6 h and cell viability was determined by MTT assay.
